# How to Quantify Penile Corpus Cavernosum Structures with Histomorphometry: Comparison of Two Methods

**DOI:** 10.1155/2015/832156

**Published:** 2015-08-27

**Authors:** Bruno Felix-Patrício, Diogo Benchimol De Souza, Bianca Martins Gregório, Waldemar Silva Costa, Francisco José Sampaio

**Affiliations:** ^1^Urogenital Research Unit, State University of Rio de Janeiro, Boulevard 28 de Setembro, 87 Fundos, Vila Isabel, 20551-030 Rio de Janeiro, RJ, Brazil; ^2^Institute for Humanities and Health, Federal Fluminense University, Rua Recife, s/n, Jardim Bela Vista, Rio das Ostras, RJ, Brazil

## Abstract

The use of morphometrical tools in biomedical research permits the accurate comparison of specimens subjected to different conditions, and the surface density of structures is commonly used for this purpose. The traditional point-counting method is reliable but time-consuming, with computer-aided methods being proposed as an alternative. The aim of this study was to compare the surface density data of penile corpus cavernosum trabecular smooth muscle in different groups of rats, measured by two observers using the point-counting or color-based segmentation method. Ten normotensive and 10 hypertensive male rats were used in this study. Rat penises were processed to obtain smooth muscle immunostained histological slices and photomicrographs captured for analysis. The smooth muscle surface density was measured in both groups by two different observers by the point-counting method and by the color-based segmentation method. Hypertensive rats showed an increase in smooth muscle surface density by the two methods, and no difference was found between the results of the two observers. However, surface density values were higher by the point-counting method. The use of either method did not influence the final interpretation of the results, and both proved to have adequate reproducibility. However, as differences were found between the two methods, results obtained by either method should not be compared.

## 1. Introduction

Cell and tissue morphological alterations are highly associated with functional and developmental changes and thus are the focus of scientific research [1]. Traditionally, tissue or cell morphology is studied by describing normal and/or pathological findings of the organ of interest.

Although the description of morphological changes is valid in some specific circumstances, for most situations, scientific data gain value when expressed numerically [2, 3], and this is the premise supporting the use of morphometry for medical research. Commonly, it is possible to quantify morphological changes in disease-affected structures or after medical or surgical treatments [4]. Macroscopic or microscopic quantification of a structure increases accuracy by generating numerical data that can be used for statistical comparisons, thus giving credibility to the study [5, 6].

Score quantification based on observer's interpretation has been previously reported, but as the result depends directly on experience, the method is less reliable and reproducible. Thus, the use of objective morphometric analytical methods is preferable, as observer's experience will have little impact on the outcome of the results [5, 6].

The quantification of structure surface density of an organ, tissue, or cell allows their characterization and comparison in different pathological conditions. For example, Bertoni-Freddari et al. [7] measured synaptic surface density in different areas of the cerebral cortex of monkeys at different ages to study the effects of aging on the brain, while Romek et al. [8] used this methodology to study inner mitochondrial membrane surface density in relation to the preimplantation development and metabolic alterations of the porcine embryo. Therefore, these methods provide important information to enable comparisons between groups of patients or animals subjected to different conditions.

The surface density of a structure of interest is traditionally calculated using the point-counting method, whereby the number of points that intercepts the structure is divided by the total number of points superimposed to the field of interest. Although this method is considerably reliable, as long as its premises (i.e., randomization, repetition, and blind measurements) are respected [5, 6, 9–11], the time spent counting the points of each analyzed image is a major disadvantage.

Computer-aided quantification of structures based on the area occupied by certain colors has emerged as an alternative [12–14]. This method, called color-based segmentation, requires less time for its execution, accelerating the achievement of scientific results. According to this method, the structure to be quantified must have a color distinguishable from the other structures in the image, so that the computer can measure the percentage of the area occupied by that color, and consequently of the structure of interest.

Although both methods are considered reliable and reproducible for surface density determination, they have not been objectively compared under the same biological conditions. In principle, comparable structure surface density results should be obtained using the point-counting or color-based segmentation method in the same group of individual samples.

The aim of this study is to compare the results of surface density analysis of penile corpus cavernosum smooth muscle in different groups of rats, measured by the point-counting and color-based segmentation method and by two different observers.

## 2. Materials and Methods

### 2.1. Experimental Design

Twenty 120-day-old male rats were used in this experiment. Rats were maintained in an animal facility room at a temperature of 21 ± 1°C, with a controlled 12-hour light/dark cycle (artificial light, 7:00 am to 7:00 pm), and received commercial food and water* ad libitum*. All procedures were carried out in conformity with the conventional guidelines on animal experimentation. Experimental protocols were approved by the Institutional Animal Experimentation Ethics Committee (Protocol no. CEUA/051/2012).

The animals were divided into two groups: a normotensive Wistar Kyoto strain (WKY) group and a spontaneously hypertensive strain (SHR) group, containing 10 rats each. Systolic blood pressure was measured weekly to validate the experimental models used [15].

### 2.2. Euthanasia and Histological Procedures

**Figure 1:**
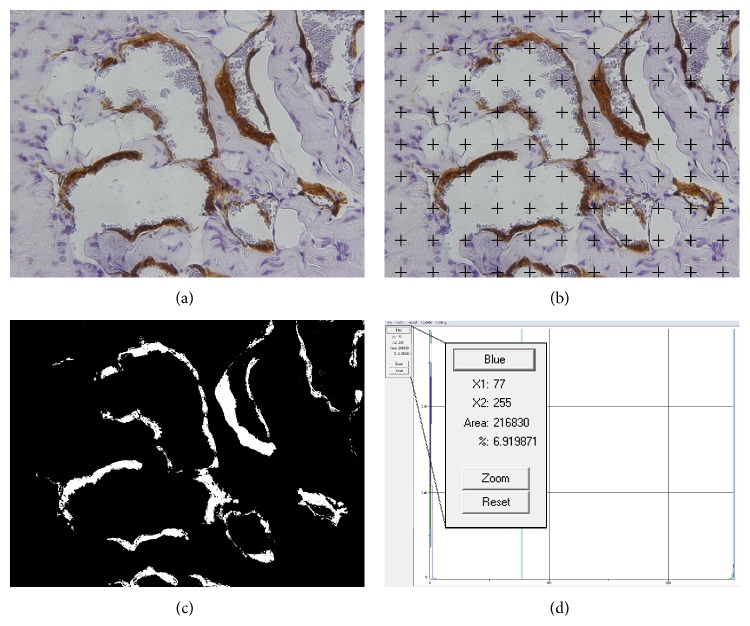
(a) Example of a histological field of a rat's corpus cavernosum immunostained with antismooth muscle *α*-actin and captured under a ×400 magnification field. (b) The same field after superimposition of the 99-point grid. The points touching the smooth muscle were counted. (c) The same field after all smooth muscle areas was transformed into white colored pixels while the remaining pixels of the images appear in black. (d) Histogram data of image (c) showing that 6.9% of the image is composed by white pixels, that is, smooth muscle.

Rats were euthanized at 160 days of age with an anesthetic overdose and their penises were dissected and fixed in 4% buffered formalin. Because of the presence of a distal bone in the rat penis, the midshaft of each organ was used for morphological analyses. This tissue was processed for paraffin embedding and 5 *μ*m thick sections were prepared. Immunolabeling was performed using a primary antibody antismooth muscle *α*-actin (Zymed Laboratories, Carlsbad, California) (Figure 1(a)).

All morphometrical analyses were carried out from photomicrographs captured under ×400 magnification, using a digital camera (DP70, Olympus, Tokyo, Japan) coupled to a microscope (BX51, Olympus, Tokyo, Japan). For each animal, 25 histological fields of cavernous tissue were photographed. In these photomicrographs, the trabecular smooth muscle surface density was quantified by the two different methods described below. All morphometrical analyses were performed by two different researchers.

### 2.3. Counting-Point Method [4, 9, 16–18]

For this analysis, the Image J software (version 1.45s, National Institutes of Health, Bethesda, USA) was used. A 99-point grid was superimposed over the images using the grid tool of Image J software, and the points touching the trabecular smooth muscle were marked and counted with the cell counter tool. The number of points touching the smooth muscle was multiplied by 100 and divided by 99 to correct for the 99 points used as test system (Figure 1(b)). This result was considered the surface density and expressed as a percentage. The mean of 25 analyzed photomicrographs was considered as the smooth muscle surface density for each animal.

### 2.4. Color-Based Segmentation [13, 19, 20]

For this analysis, the Image-Pro Plus software (version 4.5.0.29z, Media Cybernetics, Rockville, USA) was used. The smooth muscle surface area was calculated using the histogram tool after a color segmentation of the image, based on automatic counting of the percentage of pixels with the same color (brown in our immunohistochemistry images).

First, the brown colored pixels in the image were marked with the perform segmentation tool, selecting different positive stained areas, and a mask created with the new mask tool. All selected areas of the images were transformed into white colored pixels while the remaining pixels appeared in black (Figure 1(c)). Then, the “histogram” tool was opened and moving the bar to the right, the percentage of white pixels was determined by the software (Figure 1(d)). This percentage represents the surface density of brown colored areas, previously selected, and, thus, the smooth muscle surface density. The mean of 25 analyzed photomicrographs was considered as the smooth muscle surface density for each animal.

### 2.5. Statistical Analysis

The data were first tested for normality using the Shapiro-Wilk normality test. All data passed the normality test and were considered to be parametric (*P* > 0.05). The means of the WKY versus SHR group, obtained by each observer and analyzed by the counting-point and color-based segmentation methods, were compared using an unpaired Student's *t*-test. Finally, to test the reproducibility of each method, the results obtained by observers A and B were compared with the paired Student's *t*-test. All analyses were performed using the GraphPad Prism 5.0 software (GraphPad Software, San Diego, USA). Mean differences were considered significant if *P* < 0.05. All results are presented as the mean ± standard deviation.

## 3. Results

**Figure 2:**
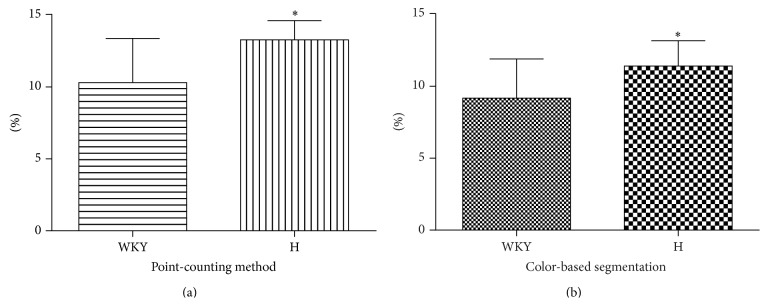
(a) Smooth muscle surface density measured by the point-counting method in the corpus cavernosum of normotensive and hypertensive rats (^∗^ *P* = 0.012). (b) Smooth muscle surface density measured by the color-based segmentation method, in corpus cavernosum of normotensive and hypertensive rats (^∗^ *P* = 0.038) (columns and error bars represent the mean and standard deviation, resp.). Results shown are those of observer A.

The smooth muscle surface density analyzed by the point-counting and color-based segmentation method was 26–29% (observer A *P* = 0.035; observer B *P* = 0.012) and 25–32% (observer A *P* = 0.038; observer B *P* = 0.002) higher, respectively, in hypertensive (SHR) than in normotensive (WKY) animals (Figure 2 and Table 1).

Comparison of smooth muscle surface density between the two methods in normotensive (WKY) animals showed no difference between the means obtained by observer A (*P* = 0.119), but the means obtained by observer B were significantly different (*P* = 0.001). Moreover, when smooth muscle surface density between the two methods was compared in the hypertensive (SHR) animals, the means obtained by the point-counting method were higher than that obtained by the color-based segmentation method, for both observers (observer A *P* = 0.001; observer B 0.002).

Finally, when comparing the results obtained by observer A and observer B, for each group of animals and type of method, no statistical differences were found (point-counting method, WKY *P* = 0.437; SHR *P* = 0.323; color-based segmentation method, WKY *P* = 0.180; SHR *P* = 0.518).

## 4. Discussion

The quantification of morphological structures is highly recommended for studying biological alterations in tissues, cells, or intracellular organelles. Translating the morphology in numbers is useful as it improves the understanding of changes in the specimens under examination [6, 11]. In addition, numerical data allows statistical comparisons with other specimens, subjected to different conditions or at different developmental stages. Based on these premises, morphometry has been extensively used in different biomedical research fields, and important scientific knowledge has been generated from morphometrical analyses [21–24].

Surface density is one of the most commonly used morphometrical tools. It represents the percentage of area occupied by the measured structure, which according to the Delesse principle, allows its quantity estimation [5, 9]. Given the importance of surface density measurement, we decided to study this tool more thoroughly.

However, as intra- and interobserver variability (the normal biological variation) in the quantification of the same structure occur, the number of measurements necessary for the adequate estimation of surface density needs to be taken into account. One principle used for the determination of the number of measurements is that the structure being measured by the point-counting method should be touched by 200 points for each individual [11]. Accordingly, it is thought that 20 fields in which 99 points are counted in each should be sufficient to measure the smooth muscle rat's corpus cavernosum. In our studies of cavernous tissue in different species, surface density measurements are commonly performed by counting 99 or 100 points per field, in 25 fields. In this study, we used our standard laboratory protocol and analyzed 25 fields per animal. Considering that 10 animals were studied per group, we analyzed 250 fields or 24,750 points.

However, calculating surface density by the point-counting method is time-consuming, as the analysis of the structure of interest requires the full observer's attention to avoid overcounting or missing points that touch the studied structure. To overcome these issues, the color-based segmentation method is increasingly being used in several laboratories (as used in this study or with some variations) [13, 14, 19]. This method is based on the principle of differential color staining of structures, after which the percentage of pixels of one color can be measured in the field using different image editing software. This measurement can be performed in a few steps and the final results are rapidly obtained. Moreover, compared to the point-counting method whereby only some samples of the images (where the superimposed points are located) are analyzed, the color-based segmentation method allows the analysis of the whole field. These features highly increase precision and favor the use of the color-based segmentation over the point-counting method.

However, the great advantage of the so-called automated methods is the absence of observer's interference. As mentioned above, a distraction may result in a researcher missing or overcounting the points touching the structure of interest, leading to underestimation or overestimation of surface density when the point-counting method is used. In principle, these types of error should not occur with automated methods as the analysis is not interpreted by an individual. Nevertheless, discrepancies between different tones of the same color may not be automatically adjusted by the software, resulting in a lack of color uniformity within the same image and in different images. For example, the immunohistochemical preparations shown in this study appeared to have different tones of brown, most of them corresponding to smooth muscle. Thus, to reduce color variation, manual adjustment by the observer is required. However, this not only adds bias due to systematical over- or underestimation of surface density structure but also increases the time it takes for the researcher to perform the analysis. Thus, special attention should be taken when using this color adjustment tool.

In this study, the interpretation of results was not affected by the method used to measure cavernous smooth muscle surface density. This parameter showed a statistically significant increase in hypertensive animals, comparable between the two methods (25% versus 29%). Also, it was found that the results obtained with both methods were reproducible as no differences were observed between observers. This is a very important aspect to consider, since reproducibility is one of the pillars of morphometric evaluation of biologic structures.

It is possible that when the point-counting method was used, some points that did not touch the smooth muscle were counted as such, leading to count overestimation. However, it is more acceptable that when the color-based segmentation method was used a common mistake has occurred. When setting the software to interpret what tones should be considered brown, some dark or light brown shades could be missed, resulting in count underestimation.

However, as color standardization in all fields is challenging, color tone differences are common in histological images. These issues hinder the application of the color-based segmentation method to histological specimens and should be taken into consideration when choosing the method. This study showed that, although the interpretation of results was not affected, differences between methods were observed. Compared to the systematic errors to which the color-based segmentation method is prone regardless of researcher's experience, we favor the use of the point-counting method, despite being time-consuming, as a well-trained researcher is less likely to make counting errors.

## 5. Conclusion

The use of the point-counting or color-based segmentation method did not influence the final interpretation of results, and both proved to be reproducible between different researchers. However, as differences were found between the two methods, results obtained by either method should not be compared.

## Figures and Tables

**Table 1:** Smooth muscle surface density of corpus cavernosum of Wistar Kyoto normotensive animals (WKY) and spontaneously hypertensive rats (H) as measured by two different morphometrical methods.

Observer	WKY	H	*P* value
A			
Point-counting method (%)	10.30 ± 3.08	13.28 ± 1.33	0.012
Color-based segmentation method (%)	09.18 ± 2.72	11.48 ± 1.76	0.038

*P* value	0.119	0.001	

B			
Point-counting method (%)	11.08 ± 2.27	13.94 ± 1.45	0.003
Color-based segmentation method (%)	07.85 ± 0.93	10.83 ± 2.34	0.002

*P* value	0.001	0.002	

Data are shown as mean ± standard deviation. Means were considered significantly different if *P* < 0.05.
